# Readability and Content Assessment of Informed Consent Forms for Medical Procedures in Croatia

**DOI:** 10.1371/journal.pone.0138017

**Published:** 2015-09-16

**Authors:** Luka Vučemilo, Ana Borovečki

**Affiliations:** 1 Department of Otorhinolaryngology, University Hospital Merkur, Zagreb, Croatia; 2 University of Zagreb, School of Medicine, Andrija Štampar School of Public Health, Zagreb, Croatia; Institute of Tropical Medicine (NEKKEN), Nagasaki University, JAPAN

## Abstract

**Background:**

High quality of informed consent form is essential for adequate information transfer between physicians and patients. Current status of medical procedure consent forms in clinical practice in Croatia specifically in terms of the readability and the content is unknown. The aim of this study was to assess the readability and the content of informed consent forms for diagnostic and therapeutic procedures used with patients in Croatia.

**Methods:**

52 informed consent forms from six Croatian hospitals on the secondary and tertiary health-care level were tested for reading difficulty using Simple Measure of Gobbledygook (SMOG) formula adjusted for Croatian language and for qualitative analysis of the content.

**Results:**

The averaged SMOG grade of analyzed informed consent forms was 13.25 (SD 1.59, range 10–19). Content analysis revealed that informed consent forms included description of risks in 96% of the cases, benefits in 81%, description of procedures in 78%, alternatives in 52%, risks and benefits of alternatives in 17% and risks and benefits of not receiving treatment or undergoing procedures in 13%.

**Conclusions:**

Readability of evaluated informed consent forms is not appropriate for the general population in Croatia. The content of the forms failed to include in high proportion of the cases description of alternatives, risks and benefits of alternatives, as well as risks and benefits of not receiving treatments or undergoing procedures. Data obtained from this research could help in development and improvement of informed consent forms in Croatia especially now when Croatian hospitals are undergoing the process of accreditation.

## Introduction

Informed consent is process whereby patients express their consent or refusal to medical intervention based on information provided by a health care professional regarding the nature and potential consequences of the proposed medical intervention [1]. In Croatia according to the Act on the Protection of Patients’ Rights patient expresses acceptance of certain diagnostic or therapeutic procedure by signing the informed consent form [2]. Informed consent forms in Croatia consist of two parts: the statement of acceptance or refusal, and the written information part that provides patient with information regarding recommended diagnostic or therapeutic procedure [3]. The part dealing with the statement of acceptance or refusal of recommended medical procedure is regulated by the ordinance of the Ministry of Health and Social Welfare from 2008 and contains: general data about patient, medical institution, and a signature of patient or guardian and signature of physician [3]. However, the content of the written information part that provides patient with information regarding recommended diagnostic or therapeutic procedure is not defined by the ordinance. This part is left to be drafted by each medical institution with the prior opinion of the medical chamber and with the approval of the Agency for Quality and Accreditation in Health Care and Social Welfare [3].

According to the Act on the Protection of Patients’ Rights the patients have the right to get information in a way that is understandable considering their age, education level and mental ability [2], and this should be applied to both verbal and written information. Given that the patients in the hospital receive written information, including informed consent forms, it is essential that materials for patients be written in an understandable way [4]. If patients cannot read or comprehend written materials provided to them, and if this information is not sufficient, its purpose will be of limited use [5,6]. Consequently to the above, numerous studies assessing readability show that written materials for patients are written at levels beyond the patients' literacy level [7,8]. Assessed Web sites and online materials for specific medical conditions or specific diagnostic or therapeutic interventions contain online medical information of which readability levels exceed recommended reading levels of the average adult population [4–6]. The same pattern is observed with the readability of informed consent forms [9,10].

Health literacy is linked to literacy and entails people’s knowledge, motivation and competences to access, understand, appraise, and apply health information in order to make judgments and make decisions in everyday life concerning healthcare, disease prevention and health promotion to maintain or improve quality of life during the life course [11]. The data from the European Health Literacy Survey show that nearly half the Europeans surveyed have inadequate or problematic health literacy [12]. Inadequate health literacy has numerous consequences such as poorer health status, poorer health outcomes and poorer use of health care services [13]. Low literacy may affect decision-making process [14], and compliance of patients [7].

Overarching goal of this study was to assess the current status of medical procedure consent forms in everyday clinical practice in Croatia specifically in terms of the readability and the content of informed consent forms since the high quality of informed consent forms is essential for adequate information transfer between physicians and patients. Literature review did not establish studies that would examine the readability of informed consent forms written in Croatian language using readability formulas adjusted for Croatian language [15]. Before now there were no studies that assessed content of informed consent forms in Croatia.

## Materials and Methods

We have collected 55 different informed consent forms from six hospitals in Croatia both on the secondary and tertiary health-care level. Forms were collected as part of the extensive research about the patients’ right to information in the Croatian hospitals. Hospitals were randomly selected based on the national list of hospitals taking into account the geographical distribution of Croatia in 6 geographical statistical regions (one hospital from each geographical statistical region). Hospitals were divided in two groups: university hospitals that are highly specialised teaching hospitals and general regional hospitals. First group consisted of University Hospital Centre Zagreb, University Hospital Centre Rijeka, and University Hospital Merkur from Zagreb. General hospitals included in this study were: General Hospital “Dr. Josip Benčević” from Slavonski Brod, General Hospital Zadar and General Hospital Zabok. In each hospital we selected 5 departments by using computer program for randomization. Prior to randomization paediatric departments, psychiatric departments, and intensive care units were excluded. All available informed consent forms from the departments that were included in the survey were collected. 52 of the forms consisted of written patient information part explaining different diagnostic or therapeutic procedures and of the part which required patient’s signature as required by the ordinance. Three documents we labeled as statements because they contained only general statement about accepting or refusing medical procedure without any information about medical procedure and these were excluded from analysis.

### Readability assessment

All the materials were in Croatian language thus for assessment of readability of collected informed consent forms we used Simple Measure of Gobbledygook (SMOG) formula adjusted for Croatian language in 2011 by Brangan [16]. SMOG measures difficulty of content by the number of polysyllabic words and score or SMOG grade is presented as the reading grade to indicate the education level required to understand the given text [17]. SMOG was used because of its high frequency of use in the assessment of the health literature [4–9,18], its accuracy and its high correlation with other readability formulas [19]. For our analysis polysyllabic words were considered to be those words in Croatian language that have four or more syllables [16], while in the English formula polysyllabic words are those that have three or more syllables [17]. Reason for using four or more syllables in analysis is because of fact that in the Croatian language words are on average longer by number of syllables than in the English language [16]. There are no computer programs with readability formulas adjusted for Croatian language so all calculations were done manually. We omitted bulleted and numbered lists, and headings and sub-headings. Text within tables was analyzed in cases when content was formed as sentences. If the informed consent forms had more than 30 sentences we would take ten consecutive sentences near the beginning, in the middle, and near the end of the document, for a total 30 sentences [17]. Then, total number of polysyllabic words within those sentences was counted and the square root of the nearest perfect square was obtained. The number two (in Croatian adjusted formula) was added to the integer to obtain the grade level of the document [16,17]. If the informed consent form had fewer than 30 sentences all sentences were included in analysis and a modified formula was used. In this case, all polysyllabic words were counted in the text. After that the average number of polysyllabic words per sentence was counted and multiplied by the number of sentences short to 30. This figure was added to the total number of polysyllabic words. Then, the square root was calculated and the constant of two was added as in longer text [16]. Readability formulas are recommended here as rough estimates of the difficulty of written materials for patients since reading tests applied to test comprehension are not applicable for Croatian language because of its orthographic transparency, and readability formulas correlate well with comprehension tests [16].

### Content assessment

To analyze content of informed consent forms a total 11 items was evaluated in each informed consent form using a checklist. For our analysis we modified Checklist for assessing the Informed Consent Form of Temple University Health System [20]. We divided items in two groups: basic elements and general data. Basic elements of informed consent forms were: information about procedure, risks, benefits, alternatives, risks and benefits of alternatives, risks and benefits of not receiving treatment or undergoing procedures. General data that was considered to be: name and signature of patient, name and signature of physician, name of hospital, date of procedure, and statement that procedure was explained.

Two reviewers (LV, NV) rated informed consent forms independently and disagreements were resolved by consensus.

For statistical analysis we performed descriptive statistics (means, standard deviations, percentages) and the t-test using Microsoft Excel (version 2007) while our data have normal distribution. Significance was set as p< 0.05.

Ethics approval was obtained from the Ethics Committee of the School of Medicine, University of Zagreb and from the Ethics Committees of each hospital.

## Results

**Fig 1:**
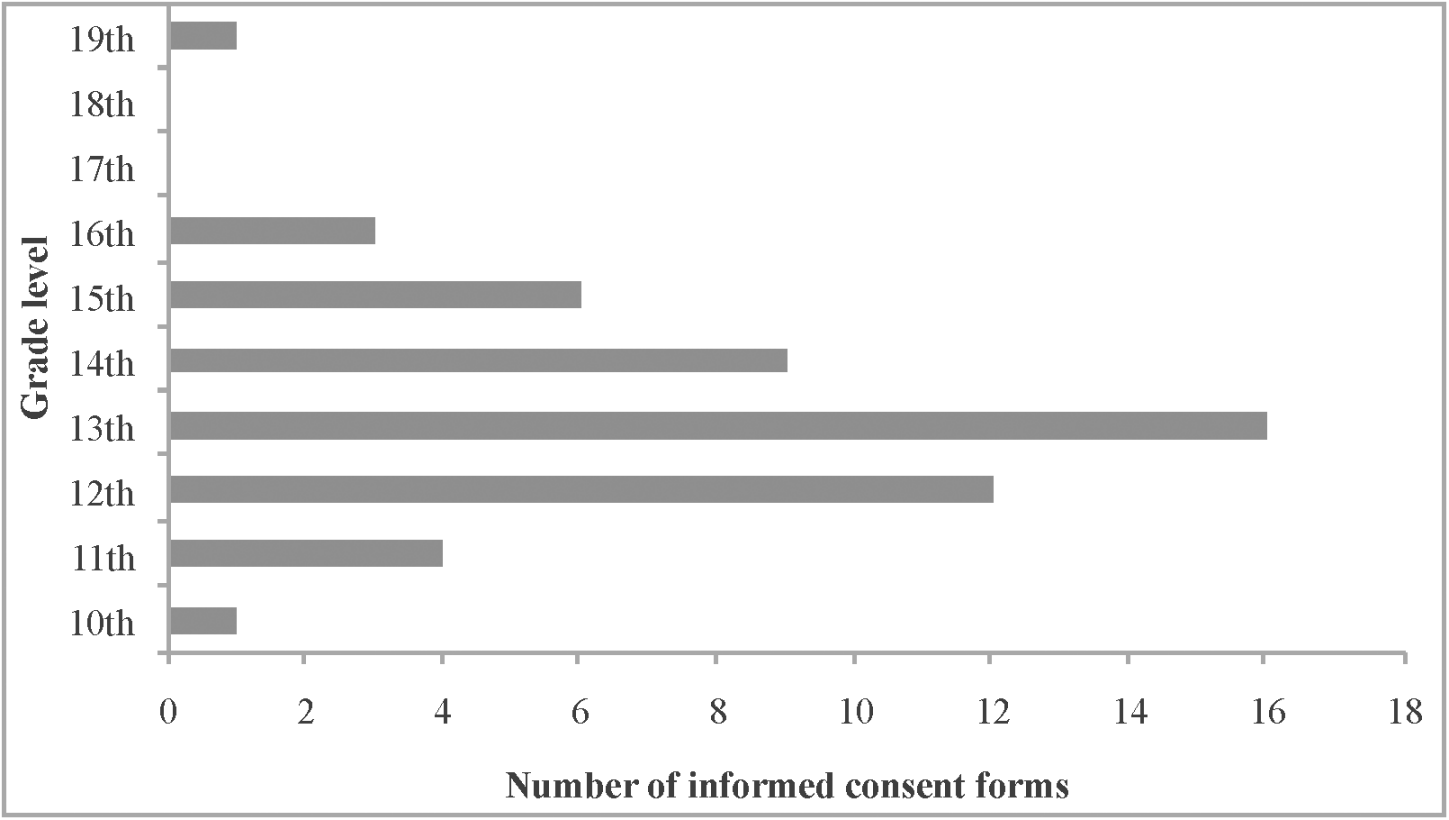
Distribution of analyzed informed consent forms by grade level.

Mean readability of informed consent forms was grade 13.25±1.59 using SMOG (range 10–19). There was no informed consent form written below the tenth grade level (Fig 1). 7.7% informed consent forms were written at or beyond the sixteenth grade level. Twenty nine of 52 collected informed consent forms were from the general hospitals on the secondary health-care level. There was no significant difference between informed consent forms prepared in the Croatian hospitals at the secondary (13.07±1.22) to those used in hospitals on the tertiary health-care level (13.48±1.97) (p = 0.405). Those informed consent forms that were labeled as therapeutic consents forms had higher readability SMOG score than those that were labeled as diagnostic consent forms: 13.64±1.65 vs. 12.89±1.48, although without significant difference (p = 0.091). No difference was observed in the readability level when consent forms containing more than 30 sentences (13.33±1.05) were compared with shorter ones (13.18±1.96), (p = 0.719). There was no significant difference between consent forms that are used by internists (13.15±1.56) in comparison to those used by surgeons (13.36±1.73), (p = 0.653).

Eleven analyzed content items of informed consent forms are listed in Table 1 where is also presented the number of informed consent forms that included these content items. Only two informed consent forms (3.8%) had all analyzed elements. Five of six basic elements were included in 19.2% of all informed consent forms, four of six elements in 15.4%, three of six in 36.5%, two of six in 19.2%. 5.7% of all informed consent forms had none or one basic element included. It was also observed that informed consent forms that have more than 30 sentences have in average 3.96±1.16 basic elements included in the content, while informed consent forms shorter than 30 sentences have in average 2.78±1.26 basic elements (p = 0.001). There was no statistical difference when content of informed consent forms was compared with level of health care institution, secondary (3.21±1.18) or tertiary level (3.48±1.53), (p = 0.487), with type of informed consent forms, diagnostic (3.44±1.19) or therapeutic (3.20±1.50), (p = 0.520), with provider of information, internists (3.33±1.21) or surgeons (3.18±1.52), (p = 0.969). Informed consent form page numbers ranged 1 to 6, (median 2.50).

**Table 1:** Distribution of content items of 52 analyzed informed consent forms.

**Basic elements**	**Number (%)**
Information about procedure	41 (78.8)
Risks	50 (96.1)
Benefits	42 (80.8)
Alternatives	27 (51.9)
Risks and benefits of alternatives	9 (17.3)
Risks and benefits of not receiving treatment or undergoing procedures	7 (13.5)
**General data**	
Name and signature of patient	39 (75.0)
Name and signature of physician	41 (78.8)
Name of hospital	51 (98.1)
Date	51 (98.1)
Statement that procedure was explained	41 (78.8)

## Discussion

Our data have shown that mean readability of evaluated informed consent forms was grade 13.25 using SMOG. SMOG grades 13 and above indicate the need for college education [17]. This further indicates that the content of informed consent forms writing is complex and difficult to read for the general population. Our results correspond to results of other authors that performed readability assessment of informed consent forms written in English and Spanish [9,10] or used other readability formulas [9,10,21]. High scores raise concerns about whether patients understand informed consent forms they are provided with. This may pose some serious problems to informed consent procedures in Croatian hospitals especially if we consider that according to the 2011 Croatian census 1.7% of adult Croats have no formal education, 29% of Croatian population has primary school level of education and 52% of population has secondary level of education [22]. Primary education lasts in the Croatia 8 years, secondary education lasts additional 4 years, academic education at least additional 3–5 years, in some cases if we count PhD academic education lasts 9–10 years. This means more than 80% of the Croatian people older than 15 years has less than 13 years of education (primary and secondary education) and would not be able to understand informed consent forms that are now in use in Croatian hospitals. There was no research done in Croatia on the level of health literacy on the national level while health-literacy-assessment tools as the Rapid Estimate of Adult Literacy in Medicine and the Test of Functional Health Literacy in Adults are not adjusted for Croatian language [16]. In Europe, when it comes to health literacy on average 47% persons have inadequate or problematic health literacy level [12]. The range of health literacy varies form 29% of respondents with limited health literacy in Netherlands to 62% in Bulgaria [12]. Available studies also confirm that informed consent forms are usually written in such a way that patients frequently do not understand information disclosed to them [9,10]. Our results show that none of assessed informed consent forms were written for the level of primary school below the eighth grade although health information for the general population in Croatia should be written for the level of the fifth or sixth grade [16].

Specific content criteria were found to be absent from many informed consent forms [9]. Our analysis of content of informed consent forms showed omissions in specifying to the patients risks and benefits of alternative treatments and procedures, as well as omissions in informing patients about risks and benefits of not receiving treatment or undergoing procedure. Some authors found that the major problem with informed consent forms had to do with lack of specifying benefits and opportunities of a treatment or procedure to the patient, and inadequate facilitation of a patient-provider interaction [9]. For other authors the lack of clear explanation of risks connected to certain treatments and procedures was the major problem observed [8]. Moreover, Bottrell et al [23], analyzed four basic elements of informed consent forms, which included nature of the procedure, risks, benefits, and alternatives. They have concluded that the content of the most forms did not meet accepted standards [23]. Readability assessments of the different content areas of informed consent forms could not be achieved in our study because only two of 52 informed consent forms have all basic elements included and different content areas are not suitable for performing SMOG analysis due to text brevity. Croatian informed consent forms as shown by our analysis lack some basic elements. This may be connected to the fact that there is no law, ordinance or guidance that clearly defines all the necessary elements of content of informed consent forms. Similar situation is observed in Nigeria where the scant content of informed consent forms was reported in the absence of any guideline [21]. In Croatia the Ministry of Health gave only directions for general data that informed consent forms are required to contain [3]. Our results indicate that general data, which are regulated with ordinance, are in greater percentage included in the content of informed consent forms. Therefore, due to absence of guidelines, all parties involved in creation of informed consent forms face difficulties with their drafting. Decision is left to each hospital and each department to create their own informed consent forms for the same medical procedures. Internationally, different professional organizations create guidelines for creation of informed consent forms [20,24]. In these countries another problem may be observed that the content, which is required to appear in informed consent documents as stipulated by regulating bodies, creates a barrier to developing consent forms at recommended eighth grade reading level or lower [25]. Creating easy to read informed consent forms presents a challenge even for the very mindful and skilled writers who need to meet federal requirements and professional guidelines [25].

Additional problem observed in Croatia is that there is no national list of medical procedures that require written informed consent forms. This decision is also left to each department and each hospital [15]. Important aspect of health care, such as informed consent form in everyday clinical practice, should not depend on individual interpretation of what informed consent is and what it should represent to patients. Common situation in the Croatian hospitals is that patients during hospital admission sign general consent form that contains only general statement about accepting or refusing medical procedure without any information about medical procedure [26]. Additionally, patients may sign consent form for specific medical procedure, such as anesthesia, blood transfusion, radiological examinations, particular invasive procedures, but frequently patients do not sign anything else since there are no informed consent forms developed for all those specific procedures in certain institutions.

One of the patients’ rights is right to be fully informed and to receive information in a way that is understandable considering the education [2]. If patients do not understand written information or if information is not fully disclosed to them ensuring equal health care for all comes into question. Our results suggest that informed consent forms currently used in Croatian hospitals are not in adherence to the Croatian legal provisions and to the patients’ right for understandable information. Unfortunately, there are numerous examples were written materials for patients, including the consent forms, are written in a way that they do not fulfill its purpose [6,8–10]. Patients who do not receive adequate information cannot co-decide about their medical condition, which leads to diminution of active role of patients in the decision-making process. Insufficient patient information was also observed when patients received verbal information by physicians [27]. In study where physician-patient communication was explored in a hospital setting in Croatia patients reported that they were informed about health risks of the proposed treatment in 74% of cases, about health consequences of refusing a medical intervention in 69% of cases and about other methods of treatment in 46% of cases [27]. Mentioned problems affect the informed consent process directly.

Health care providers should not take for granted patients' ability to read and understand medical information. Although the readability of informed consent forms assessed in our research was not adequate, and needs to be improved, improving reading level alone will not guarantee that patients will understand or use medical materials. Recommendations when developing informed consent forms are to use simple language, shorter sentences, and active voice, avoid unnecessary words and technical jargon, and write shorter sentences to break up material, use clear headings and sub-headings, use illustrations [10,20, 25]. In examined informed consent forms there were no graphics and illustrations. The US National Cancer Institute strongly recommends that consent forms for adult clinical trials do not exceed six to nine pages [24]. Although, Denzen et al [25], consider that the priority should be given to readability over length. Nishimura et al [28], in systematic review of research informed consent concluded that enhanced consent forms and extended discussions are most effective in improving participant understanding. When developing consent form it is important to think about population for whom consent forms is intended and literacy level of patients, and to include patients to participate in drafting and deciding on the final version of the written materials for patients [20].

A limitation of this study is that our consent forms sample was convenience sample. Randomization of informed consent forms could not be achieved although departments and hospitals were randomly selected. Reason for having convenience sample was because we did not know which hospitals and departments have developed informed consent forms and for what medical procedures so we have collected all current available informed consent forms. Our advantage is that we have analyzed readability of different informed consent forms while majority of previous published papers deal with informed consent forms for specific procedure (only surgical informed consent forms, informed consent forms in cardiology, informed consent forms for radiation therapy of cervical cancer, dental informed consent forms) [9,10,29,30]. Also, there are limitations of readability formulas themselves such as not considering the influence of visual and design factors, or readers’ prior knowledge and motivation [19]. We used only one readability formula in assessment of informed consent forms because there are numerous studies using only SMOG when assessing readability of patients’ materials [5,8]. Differences in reading level scores can be found if same written materials are assessed by different readability formulas [4,18]. Higher mean scores are observed when assessing reading level using SMOG than the Flesh-Kincaid [4,18]. Estrda et al [7], recommend not using Flesch-Kincaid formula to determine readability of printed information. We did not assess accuracy of the information in the content analysis.

Authors have several recommendations to improve the informed consent process in Croatia. Ministry of Health should define national list of medical procedures that require written informed consent forms. All involved in creation of informed consent forms (the medical institutions, the patient associations, the Croatian Medical Chamber, the Agency for Quality and Accreditation in Health Care and Social Welfare) should collaborate and strive to reinforce informed consent process by contributing written materials that have all relevant information included in the content and are written at an appropriate reading level designed for comprehension by patients at all levels of health literacy. Additionally, all stakeholders in health care should develop surroundings for assessing patients understanding of and satisfaction with the consent process for medical procedures because well trained and knowledgeable consenters are also important. Enhancing informed consent forms will lead to empowerment of active patient role and enhancement of shared decision-making process, that is, to the improvement of informed consent process. We encourage the implementation of adjusted readability formula for Croatian language in assessment of different written patient materials. We hope that our research will instigate the research of informed consent forms using same approach in other countries whose languages bare similarities to Croatian language.

## Conclusions

There is place for improvement of the content and readability of informed consent forms in Croatia. Evaluated informed consent forms were written for an educational and reading level considerably higher than the level of the majority of Croatian population. The content of the forms failed to include in high proportion of the cases description of alternative treatments and procedures and benefits of alternative treatments and procedures, as well as risks and benefits of not receiving treatments or undergoing procedures. Data obtained from this research could help in the development and improvement of informed consent forms in Croatia especially now when Croatian hospitals are undergoing the process of accreditation, which is an opportunity for Croatia, country that does not have decade’s old entrenched consent practices, to enhance informed consent process.

## References

[1] Coy JA. Autonomy-based informed consent: ethical implications for patient noncompliance. Phys Ther. 1989;69: 826–833. 2780809 10.1093/ptj/69.10.826

[2] Act on the Protection of Patients’ Rights, Official Gazette, no. 169 (2004). Croatian.

[3] Ordinance on the Consent Form, Official Gazette, no. 10 (2008). Croatian.

[4] Friedman DB, Kao EK. A comprehensive assessment of the difficulty level and cultural sensitivity of online cancer prevention resources for older minority men. Prev Chronic Dis. 2008;5:A07 18081996 PMC2248790

[5] Wallace LS, Lennon ES. American Academy of Family Physicians patient education materials: can patients read them? Fam Med. 2004;36: 571–574. 15343418

[6] Stossel LM, Segar N, Gliatto P, Fallar R, Karani R. Readability of patient education materials available at the point of care. J Gen Intern Med. 2012;27: 1165–1170. 10.1007/s11606-012-2046-0 22528620 PMC3514986

[7] Estrada CA, Hryniewicz MM, Higgs VB, Collins C, Byrd JC. Anticoagulant patient information material is written at high readability levels. Stroke. 2003;31: 2966–2970. 10.1161/01.str.31.12.2966 11108757

[8] Brown H, Ramchandani M, Gillow JT, Tsaloumas MD. Are patient information leaflets contributing to informed consent for cataract surgery? J Med Ethics. 2004;30: 218–220. 15082822 10.1136/jme.2003.003723 PMC1733823

[9] Glick A, Taylor D, Valenza JA, Walji MF. Assessing the content, presentation, and readability of dental informed consents. J Dent Educ. 2010;74:849–861. 20679454

[10] San Norberto EM, Gomez-Alonso D, Trigueros JM, Quiroga J, Gualis J, Vaquero C. Readability of surgical informed consent in Spain. Cir Esp. 2014;92: 201–207. 10.1016/j.ciresp.2013.02.027 24060163

[11] Sorensen K, Van den Broucke S, Fullam J, Doyle G, Pelikan J, Slonska Z, et al Health literacy and public health: a systematic review and integration of definitions and models. BMC Public Health. 2012;12: 80 10.1186/1471-2458-12-80 22276600 PMC3292515

[12] HLS-EU Consortium. Comparative Report of Health Literacy in Eight EU Member States. The European Health Literacy Survey HLS-EU, 2012 Available: http://www.health-literacy.eu. Accessed 01 August 2014.

[13] Berkman ND, Sheridan SL, Donahue KE, Halpern DJ, Crotty K. Low health literacy and health outcomes: an updated systematic review. Ann Intern Med. 2011;155: 97–107. 10.7326/0003-4819-155-2-201107190-00005 21768583

[14] Kim SP, Knight SJ, Tomori C, Colella KM, Schoor RA, Shih L, et al Health literacy and shared decision making for prostate cancer patients with low socioeconomic status. Cancer Invest. 2001;19:684–691. 11577809 10.1081/cnv-100106143

[15] Vucemilo L, Babić-Bosanac S, Altarac S, Borovecki A. Pristanak obaviještenog pacijenta s posebnim osvrtom na Hrvatsku (Informed consent with special emphasis on Croatia). Lijec Vjesn. 2014;136:104–109. Croatian 24988746

[16] Brangan S. Development of SMOG-Cro readability formula for healthcare communication and patient education. Coll Antropol. 2015;39: 11–20. 26040062

[17] McLaughlin GH. SMOG grading—a new readability formula. Journal of Reading 1969: 639–646. Available: http://www.harrymclaughlin.com/SMOG.htm. Accessed 01 August 2014.

[18] Wang C, Gallo RE, Fleisher L, Miller SM. Literacy assessment of family health history tools for public health prevention. Public Health Genomics. 2011;14: 222–237. 10.1159/000273689 20090283 PMC2891255

[19] Friedman DB, Hoffman-Goetz L. A systematic review of readability and comprehension instruments used for print and web-based cancer information. Health Educ Behav. 2006;33: 352–373. 16699125 10.1177/1090198105277329

[20] Fleisher L, Raivitch S, Miller SM, Partida Y, Martin-Boyan A, Soltoff C, et al A Practical Guide to Informed Consent. Available: http://www.templehealth.org/ICTOOLKIT/html/ictoolkitpage27.html. Accessed 01 August 2014.

[21] Ezeome ER, Chuke PI, Ezeome IV. Contents and readability of currently used surgical/procedure informed consent forms in Nigerian tertiary health institutions. Niger J Clin Pract. 2011;14: 311–317. 10.4103/1119-3077.86775 22037076

[22] The Croatian Bureau of Statistics, Census of Population, Households and Dwellings 2011. Available: http://www.dzs.hr/default_e.htm. Accessed 01 August 2014.

[23] Bottrell MM, Alpert H, Fischbach RL, Emanuel LL. Hospital informed consent for procedure forms: facilitating quality patient-physician interaction. Arch Surg. 2000;135: 26–33. 10636343 10.1001/archsurg.135.1.26

[24] National Cancer Institute. NCI Informed Consent Template–version May 12, 2013. Available: http://ctep.cancer.gov/protocolDevelopment/informed_consent.htm. Accessed 22 July 2015.

[25] Denzen EM, Santibáñez ME, Moore H, Foley A, Gersten ID, Gurgol C, et al Easy-to-read informed consent forms for hematopoietic cell transplantation clinical trials. Biol Blood Marrow Transplant. 2012;18: 183–189. 10.1016/j.bbmt.2011.07.022 21806948 PMC3242929

[26] Vučemilo L, Borovečki A, Informed consent in Croatia: a work in progress. Camb Q Healthc Ethics. 2014;23: 356–360. 10.1017/S0963180113000972 24865264

[27] Vučemilo L, Ćurković M, Milošević M, Mustajbegović J, Borovečki A. Are physician-patient communication practices slowly changing in Croatia?–a cross-sectional questionnaire study. Croat Med J. 2013;54: 185–191. 23630146 10.3325/cmj.2013.54.185 PMC3641876

[28] Nishimura A, Carey J, Erwin PJ, Tilburt JC, Murad MH, McCormick JB. Improving understanding in the research informed consent process: a systematic review of 54 interventions tested in randomized control trials. BMC Med Ethics. 2013;14: 28 10.1186/1472-6939-14-28 23879694 PMC3733934

[29] Terranova G, Ferro M, Carpeggiani C, Recchia V, Braga L, Semelka RC, et al Low quality and lack of clarity of current informed consent forms in cardiology: how to improve them. JACC Cardiovasc Imaging. 2012;5:649–655. Erratum in: JACC Cardiovasc Imaging. 2012;5: 1190. 10.1016/j.jcmg.2012.03.007 22698536

[30] MacDougall DS, Connor UM, Johnstone PA. Comprehensibility of patient consent forms for radiation therapy of cervical cancer. Gynecol Oncol. 2012;125: 600–603. 10.1016/j.ygyno.2012.02.030 22370598

